# Vinculin in cell–cell and cell–matrix adhesions

**DOI:** 10.1007/s00018-017-2511-3

**Published:** 2017-04-11

**Authors:** Jennifer L. Bays, Kris A. DeMali

**Affiliations:** Department of Biochemistry, University of Iowa, Iowa City, IA 52242 USA

**Keywords:** Vinculin, Integrins, Cadherins, Cell adhesion, Cell migration, Force and mechanotransduction

## Abstract

Vinculin was identified as a component of focal adhesions and adherens junctions nearly 40 years ago. Since that time, remarkable progress has been made in understanding its activation, regulation and function. Here we discuss the current understanding of the roles of vinculin in cell–cell and cell–matrix adhesions. Emphasis is placed on the how vinculin is recruited, activated and regulated. We also highlight the recent understanding of how vinculin responds to and transmits force at integrin- and cadherin-containing adhesion complexes to the cytoskeleton. Furthermore, we discuss roles of vinculin in binding to and rearranging the actin cytoskeleton.

## Introduction

In complex multicellular organisms, tissues are composed of one or more cell types. Cells of epithelial or endothelial origin adhere to neighboring cells through cell–cell contacts and connect to the underlying basement membrane via cell–matrix interactions. Both types of adhesions are critical for embryonic development, tissue remodeling, cell migration and other homeostatic processes. Dysregulation of adhesion complexes leads to a variety of diseases, such as cancer, diabetes and cardiovascular disease.

Adhesion is modulated by the engagement, clustering and turnover of adhesion receptors. Integrins are one such adhesion receptor and are prominently concentrated in matrix adhesions, such as focal complexes and focal adhesions. Focal complexes are small transient adhesions at the cell periphery, which grow in size to become focal adhesions—larger, more stable structures. In contrast, sites where cells adhere to neighboring cells are denoted adherens junctions and are enriched in transmembrane adhesion receptors known as cadherins.

The key to the function of adhesion receptors is the recruitment of proteins that link the adhesion receptor to the actin cytoskeleton. While many proteins are involved, vinculin—a cytoplasmic actin-binding protein enriched at both cell–cell and cell–matrix adhesions—is one of the best characterized. Vinculin has no enzymatic activity. It regulates adhesion by directly binding to actin, stimulating actin polymerization and recruiting actin remodeling proteins. In the absence of vinculin, cell–matrix and cell–cell adhesion are dramatically impaired, indicating vinculin plays a critical role in human physiology. In this review, we describe the current understanding of vinculin with an emphasis on its role in cell–matrix and cell–cell adhesive events.

### Vinculin and its binding partners

**Fig. 1:**
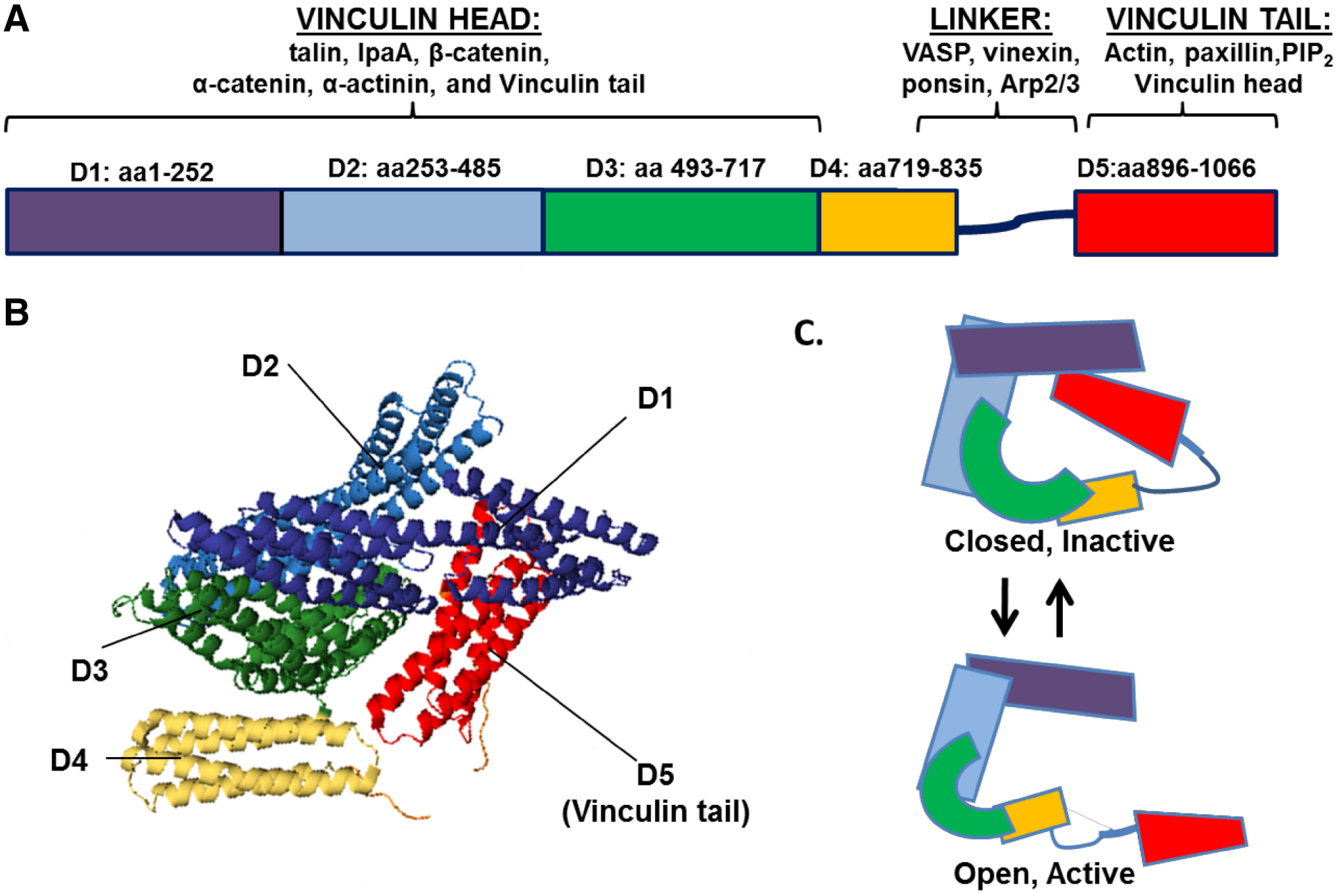
Vinculin structure and binding partners. Vinculin is comprised of anti-parallel α-helical bundles organized into five distinct domains. **a** Domains 1–3 (*D1*–*D3*) make up the vinculin head, while domain 5 (*D5*) encompasses the tail. The binding sites for many proteins interacting with vinculin have been mapped. **b** The ribbon diagram derived from the human full-length vinculin crystal structure shows vinculin resides in an inactive, closed conformation largely due to tight interactions between *D1* and *D5*. The structure was derived from the PDB coordinates that were supplied by [72]. **c** A schematic of vinculin in closed, inactive conformation and open, active conformation

Vinculin was discovered nearly 40 years ago in the laboratories of Benjamin Geiger and Keith Burridge as a 116 kDa protein highly enriched in regions where cells contact one another and the underlying substratum [1, 2]. Cloning of the cDNA revealed vinculin consists of 1066 amino acids, and subsequent crystallization studies indicated vinculin is comprised of eight anti-parallel α-helical bundles organized into five distinct domains (Fig. 1a, b) [3]. Domains 1–3 (D1-3) are arranged in a tri-lobar head with a diameter of 80 Å [4] and a molecular mass of 95 kDa [5]. The vinculin head binds many proteins including talin, IpaA, β-catenin, α-catenin and α-actinin (reviewed in [6]). Connecting the head and tail is a 61 amino acid, proline-rich linker region (residues 837–878) [3]. The linker binds vasodilator-stimulated phosphoprotein (VASP) [7], vinexin [8], ponsin [9] and Arp2/3 [10]. Lastly, the vinculin tail (amino acids 879–1066) is comprised of a 30 kDa helical bundle containing five helices connected by short loops (3–8 residues) [3, 11]. The tail contains binding sites for vinculin head domain, paxillin, acidic phospholipids and actin (Fig. 1a).

### Vinculin activation

Vinculin exists in two conformations in the cell: an open, active form and a closed, auto-inhibited state in which the head domain forms extensive interactions with the tail (Fig. 1b, c). Vinculin FRET probes that report on different conformational states show vinculin exists in its active, extended form in focal adhesions and its folded, inactive form within the cytoplasm [12].

**Fig. 2:**
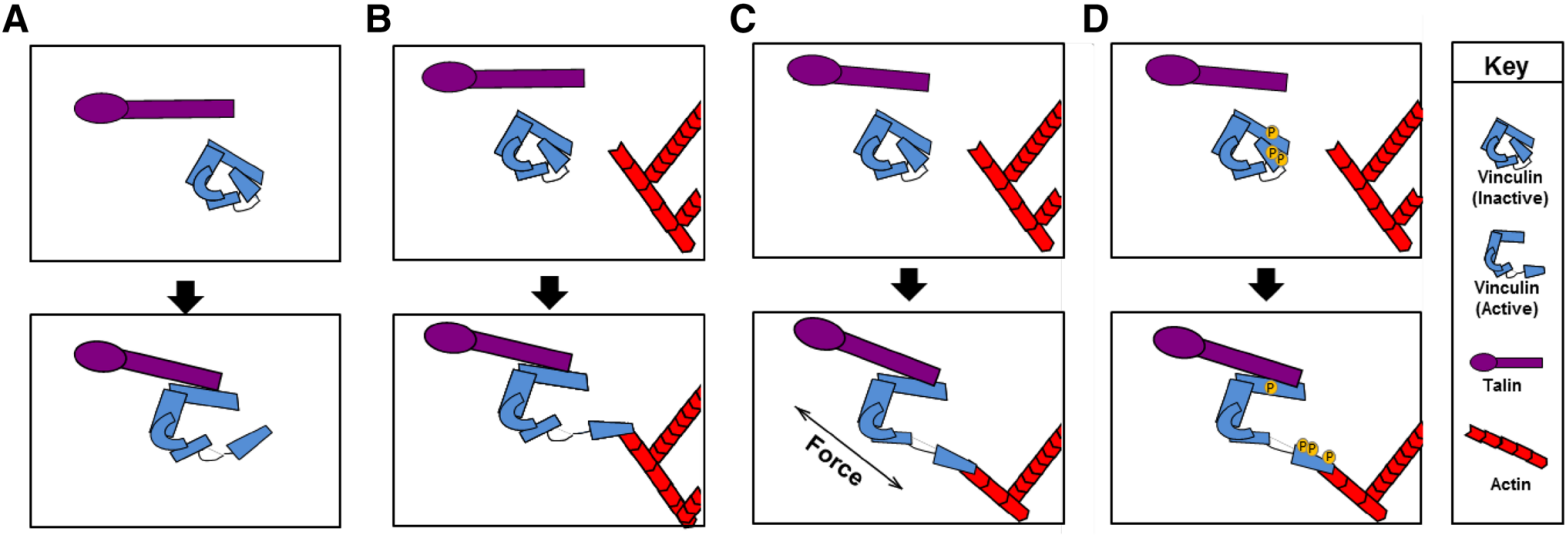
Models of vinculin activation. Vinculin exists in two conformations in the cell: an open, active form and a closed, auto-inhibited state in which the vinculin head domain interacts with the tail. Over the years, several models have been posed to explain how vinculin is opened and activated. **a** The helical bundle conversion model suggests that talin binding is sufficient to induce changes on the helical bundles in vinculin head to displace the head from vinculin tail, whereas others argue that two ligands—a head and a tail ligand—are required to separate vinculin head–tail interaction (**b**). Recent findings indicate that **c** force and **d** phosphorylation promote ligand binding and conformational changes within vinculin leading to activation

Several models have been proposed to explain how vinculin is activated within the cell. The tight binding between vinculin head and tail is thought to be too strong to be overcome by a single ligand. Indeed, the tail makes two contacts with the head and one with the linker with an overall Kd < 1 nM [3, 13]. This tight interaction led to the proposal of a combinatorial activation pathway in which two or more ligands are required to relieve the intramolecular head–tail interactions (Fig. 2). In this model, actin binding to the tail and talin, α-actinin or α-catenin to the vinculin head promotes an open conformation [14–17]. Molecular dynamic simulations have provided insight into how activation via this mechanism might occur. These studies suggest talin binds to vinculin head via surface hydrophobic interactions. This interaction allows the vinculin head domain to be freed from the tail domain and promotes conformational changes that allow talin to fully insert into the core of the vinculin head domain [19, 110].

Other evidence suggests a single ligand is enough for vinculin to adopt an open conformation. Izard et al. found talin or α-actinin binding alone induces conformation changes that displace the vinculin head from the tail in vitro, a process termed helical bundle conversion (Fig. 2) [18]. However, this model is based on studies performed using purified vinculin head domain D1 and tail. It is now recognized the vinculin head binds the tail with a 1000-fold greater affinity than the D1 domain alone [13]. Thus, activation of vinculin by a single ligand may not be achievable in the context of the full-length molecule or within the cell.

More recent studies indicate influences other than protein binding may modulate vinculin activation. For example, molecular dynamic simulations suggest phosphorylation of vinculin at Y100, Y1065, S1033 and S1045 affects activation by promoting binding of talin and actin [19, 20]. Other evidence indicates force promotes vinculin activation. In support of this assertion, force induces activating conformational changes in vinculin. Conversely, a loss of tension causes vinculin to be rapidly inactivated [21, 22]. Finally, a third possibility is phosphorylation enhances mechanical activation and vice versa [20]. Consistent with this notion, stretching uncovers tyrosine phosphorylation sites in other proteins (i.e., p130 Cas) [23, 24] as well as vinculin binding sites in talin [25]. Thus, vinculin activation is likely to be more sophisticated than the combinatorial activation or bundle inversion models predict (Fig. 2).

## Vinculin in cell–matrix adhesions

Cell–matrix adhesions or focal adhesions are rich in adhesion receptors known as integrins. More than fifty proteins are known to be recruited to the integrin cytoplasmic tail [26]. A recent investigation using super-resolution microscopy reveals these proteins are arranged in 3-D nano-domains. The identified domains are: a membrane-apposed integrin signaling layer, an actin-binding and force-transducing intermediate layer, and an uppermost actin-regulatory layer [27]. Vinculin resides within the signaling layer but is rapidly recruited to the actin-binding layer when its auto-inhibitory head–tail interactions are relieved [28]. In the actin-binding layer, vinculin facilitates the recruitment of additional proteins that regulate focal adhesion dynamics and enable efficient cell migration. Here, we discuss the roles of vinculin in cell motility. As migration is highly dependent on rearrangements of the actin cytoskeleton, this section will include a brief discussion on the relationship between vinculin and F-actin. Lastly, we will review mechanisms for regulating vinculin at focal adhesions.

### The Role of vinculin in cell migration

**Fig. 3:**
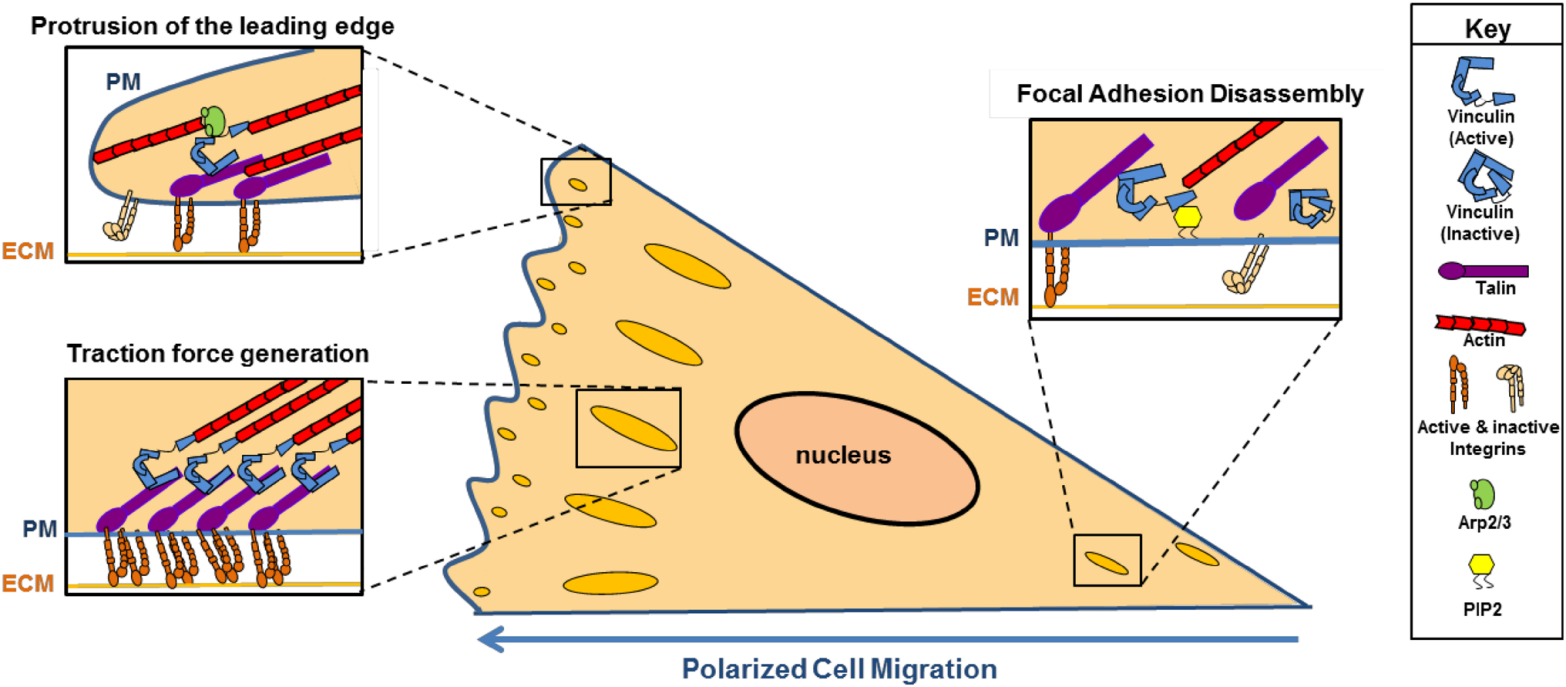
Role of vinculin in polarized cell migration. Vinculin is involved in many steps of cell migration. Its interaction with the Arp2/3 complex at nascent focal adhesions in the leading edge promotes protrusion of the membrane. During traction force generation, vinculin links integrins to the actin cytoskeleton and bears the forces exerted during motility. Lastly, vinculin interaction with PIP_2_ induces a conformation change that reduces vinculin interaction with actin, thereby promoting the disassembly of focal adhesions. *PM* plasma membrane, *ECM* extracellular matrix

Cells lacking vinculin display altered migratory properties [29]. Cell migration can be broken down into at least four steps: (1) protrusion of the leading edge, (2) adhesion to the substratum, (3) generation of traction forces to propel the cell forward and (4) breaking of older adhesions at the cell rear. In these steps, vinculin plays a role (Fig. 3).

#### Protrusion of the leading edge

In the leading edge of the cell, vinculin is localized to the first adhesions [30]. These nascent adhesions are rich in vinculin bound to the Arp2/3 complex, a potent nucleator of actin polymerization [10]. This interaction localizes actin polymerization to the newly formed adhesions [10, 31] and enables connections between integrins and actin polymerization machinery. These newly formed adhesions trigger further protrusion of the cell membrane [32]. How binding of vinculin to Arp2/3 complex is localized to the leading edge is not completely understood. There is some evidence that phosphorylation may be involved. Vinculin is highly phosphorylated at Y1065 in nascent adhesions, and phosphorylation at Y1065 regulates binding to the Arp2/3 complex [33, 34]. Moreover, vinculin deficient mutants neither recruit Arp2/3 nor support lamellipodial protrusion. Thus, tyrosine phosphorylation-regulated recruitment of Arp2/3 to vinculin is a potential mechanism to regulate membrane protrusion.

#### Adhesion to the substratum

Behind the leading edge, vinculin plays a role in maintaining focal adhesions. Indeed, early studies indicate cells overexpressing vinculin have large focal adhesions [35], while cells lacking vinculin assemble focal adhesions that are smaller and fewer in number [36, 37]. How vinculin controls focal adhesion size and number has been extensively studied. The recruitment of vinculin to talin stabilizes focal adhesions and promotes integrin clustering and enlargement [38, 39]. More recent work shows vinculin directly regulates integrin activation through talin [39–41]. Investigations into how this occurs suggest when a cell signals for increased integrin activation, talin is redistributed from the cytoplasm to the plasma membrane. Rap1-interacting molecule (RIAM) mediates the relocalization [42] and vinculin binding to talin disrupts RIAM, thereby allowing transient RIAM-positive nascent adhesions to develop into vinculin-rich, mature and stable focal adhesions [43, 44].

Interestingly, this evidence indicates talin localizes vinculin to focal adhesions. While many studies support this model, others suggest other vinculin binding partners may be involved. Specifically, talin may need assistance in settings where vinculin recruitment is rapid and robust, such as in cells under force. Paxillin may facilitate vinculin recruitment in cells under tension [45]. Indeed, myosin II activity increases paxillin binding to vinculin. Based on this information, Paspera et al. proposed a two-step “hand-off” model for vinculin recruitment to focal adhesions [45]. In this model, paxillin binds vinculin and brings it to focal adhesions. Paxillin then gives vinculin to talin [45].

In addition to modulating the activities of talin, vinculin stabilizes integrins by providing linkages to the actin cytoskeleton. How this occurs is incompletely understood. Near the leading edge of migrating cells, the vinculin tail domain captures rearward flowing actin filaments [46]. A consequence of binding to flowing actin filaments could be increased tension across vinculin. Increases in tension could promote vinculin to adopt an open conformation (as described above) and modulate actin and focal adhesion dynamics at the leading edge [46].

#### Generation of traction force

Focal adhesions near the leading edge of migrating cells transmit myosin-generated forces from the actin cytoskeleton to the extracellular matrix, thereby generating traction forces that pull the cell body forward during cell migration [47]. A critical role for vinculin in the generation of traction forces was first illustrated by studies demonstrating adhesions recruit vinculin and increase their strength in response to force [48]. More recent support of a role for vinculin in traction force generation stems from studies of migrating cells lacking vinculin in 3-D collagen matrices [36, 47]. In this setting, the loss of vinculin significantly impairs traction force generation and motility. Interestingly, while a loss of vinculin diminishes motility in 3-D matrices, mouse embryo fibroblasts lacking vinculin display increased migration rates in 2-D cultures [29]. Such evidence might indicate the lack of a role for vinculin in traction force generation. However, direct measurements of traction force generation in the vinculin null mouse embryo fibroblasts has revealed vinculin is required for traction forces in 2-D [49]. Finally, a further role for vinculin in regulating the generation of traction forces has emerged from Plotnikov et al. These investigators demonstrated that focal adhesions exhibit two types of traction force: a highly dynamic state where tugging traction forces are generated and a second state where stable traction forces are produced [50]. Vinculin is required for both types of traction forces, and both forces are required for directed cell migration. It follows that cells with decreased vinculin expression migrate more randomly than control cells [51]. Hence, vinculin plays a key role not only in the generation of traction forces, but also in directional migration of cells.

How vinculin generates traction forces is an active area of research. As initial adhesions are forming, talin is one of the first molecules recruited to integrin containing sites. At cell–matrix adhesions, talin is subject to tension which exposes binding sites for vinculin [25]. Stretching of talin induces vinculin conformational changes that reinforce F-actin anchoring, thereby allowing for the establishment of additional linkages between integrins and the actin cytoskeleton [52]. The ultimate consequence is increased integrin clustering and focal adhesion maturation [39, 53]. Therefore, vinculin promotes traction force generation by stabilizing integrin connections to the actin cytoskeleton.

#### Breaking of older adhesions at the cell rear

Given the critical roles of vinculin in regulating integrin activation and promoting the assembly of focal adhesions, it follows the disassembly of focal adhesions might require a loss or inactivation of vinculin. How vinculin is inactivated within focal adhesions is not known. There is some evidence that phosphatidylinositol 4,5-bisphosphate (PIP_2_) and calpain may be important. PIP_2_ binding induces a conformation change that reduces the vinculin tail domain from binding to actin [54, 55]. In further support of this idea, increasing the levels of PIP_2_ in cells stimulates loss of focal adhesions [54]. Another mechanism to promote focal adhesion disassembly is to interrupt vinculin–talin interactions. Calpain, a calcium-dependent protease, cleaves talin, thereby promoting focal adhesion disassembly. Mutation of the calpain cleavage site in talin renders the protease ineffective. Under these conditions, vinculin remains in focal adhesions longer and disassembly is inhibited [56]. Hence, a loss vinculin, vinculin binding to actin, or vinculin binding to talin promotes focal adhesion turnover.

### Vinculin and actin at focal adhesions

The binding of vinculin to F-actin is critical to its role at in cell–matrix adhesion. Disruptions of the vinculin-F-actin interaction affect cell morphology, cell motility, cell stiffness and adhesion [57]. The underlying cause of these defects is an impaired ability to transduce and generate forces [36, 46, 58]. In this section, we describe the current understanding for how vinculin binds F-actin and explore how this binding event may support these critical processes.

Some of the first studies of vinculin note its ability to bind F-actin [2]. However, the vinculin–actin interface has been highly debated. Originally, Janssen et al. reconstructed vinculin binding to F-actin from negative stain electron microscopy and diffraction data [15]. They predicted F-actin binds two distinct surfaces in the vinculin tail: an upper (amino acids 925–952) and lower monomer site (amino acids 1050–1056) [15]. Experimental data from four groups, including our laboratory, support residues or regions identified by Janssen are important for the interaction between vinculin and actin [13, 15, 59, 60]. However, other studies revealed that mutation of residues outside the upper and lower monomer perturb actin binding to vinculin [46, 58]. Taken together, these results suggest vinculin binding to actin is complex and likely requires multiple sites on vinculin.

In response to vinculin binding, several effects on actin have been observed. Most widely appreciated is that vinculin can cross-link and bundle actin filaments [59, 61–63]. However, vinculin can also modify existing actin bundles [64] and stimulate the formation of new bundles [64]. Additionally, vinculin can cap filaments [65] and nucleate the polymerization of new filaments [65, 66]. Lastly, vinculin can recruit actin modifiers, such as vasodilator activating phosphoprotein (an anti-capping protein) [7] and the Arp2/3 complex, an actin nucleator [10]. These properties suggest vinculin is an ideal candidate for establishing new actin assemblies and modifying existing actin structures.

### Regulation of vinculin at cell–matrix adhesions

How vinculin is regulated in cell–matrix adhesions is not fully understood. An increasing number of studies show that tension may be involved [21, 22, 45, 49, 50, 52]. Additionally, phosphorylation and oligomerization regulate vinculin function.

#### Intracellular tension

An emerging theme is that vinculin is regulated by tension. Force across vinculin stimulates focal adhesion assembly and growth [21]. It is not well understood how tension regulates vinculin activity. Tension may induce conformational changes within vinculin that could promote interactions with its binding partners [19]. Likewise, conformation changes within its binding partners could promote vinculin association. For example, force across talin stimulates unfolding of its rod domain, exposing binding sites for vinculin. Vinculin binding to talin locks talin an active conformation that stabilizes focal adhesions [67, 68]. Thus, tension-dependent conformational changes regulate vinculin binding.

#### Vinculin phosphorylation

Early studies indicate alterations in vinculin tyrosine phosphorylation correlate with a loss of cell–matrix adhesion. Later studies revealed that Y100 and Y1065 are phosphorylated during focal adhesion development and maturation by Src kinase [69]. Mutation of either of these tyrosine residues does not alter vinculin localization to focal adhesions, rather it inhibits cell spreading, migration, and the generation of traction forces [62, 70, 71].

How tyrosine phosphorylation affects vinculin function is an area of investigation. In the full-length vinculin crystal structure, Y100 is solvent exposed, whereas Y1065 is occluded by the linker domain [72]. Studies indicate Y100 and/or Y1065 are important for the binding of vinculin to phospholipids [73, 74] and actin modifying enzymes [10, 33]. Interestingly, tyrosine phosphorylation does not seem to affect vinculin binding to actin. More recent work indicates phosphorylation affects protein binding by modulating vinculin conformational changes. In support of this possibility, Y1065F or Y100F substitutions impair vinculin head tail interactions [70, 74, 75], and phosphorylation favors an active vinculin conformation [20, 70]. Molecular dynamic simulations suggest phosphorylation induces the rearrangement of charged residues in the head domain lying within the interface between D1 and tail [20]. Collectively, these studies indicate tyrosine phosphorylation at Y100 and Y1065 are important determinants of function.

Like tyrosine phosphorylation, serine phosphorylation of vinculin has also been explored. In vitro phosphopeptide mapping studies revealed two sites in the vinculin tail, S1033 and S1045, are substrates of PKC [76]. Of these, phosphorylation of S1033 is the most well studied. The Goldmann laboratory found cells expressing a phosphodeficient mutant S1033A vinculin are more pliable and less able to generate traction forces [77]. It remains speculative how phosphorylation at S1033 might affect vinculin structure. Computational simulations show that phosphorylation of S1033 could weaken vinculin head and tail interactions, thereby suggesting that serine phosphorylation regulates the conformational state of vinculin in much the same manner as tyrosine phosphorylation [20]. Validation of this idea awaits further experimentation.

#### PIP_2_ binding

Like phosphorylation, accumulating evidence suggests that phosphatidylinositol 4,5-bisphosphate (PIP_2_) may regulate vinculin function. The vinculin tail binds PIP_2_, thereby triggering conformational changes that may release the vinculin head domain to allow for talin binding [78–80]. Alternatively, PIP_2_ together with a head ligand (talin or α-actinin) and/or F-actin may be necessary for vinculin activation in vitro [3, 18, 81–83]. Finally, an ability to form oligomers may also be important. The binding of the vinculin tail to F-actin and PIP_2_ promotes oligomerization. In response to actin binding, vinculin forms dimers, and PIP_2_ triggers the formation of dimers and high-ordered structures (i.e., trimers and tetramers) [84, 85]. The physiological consequences of the various oligomeric states as well as the outcomes of PIP_2_ binding await more investigation.

## Vinculin in cell–cell junctions

Vinculin functions in cell–cell adhesions are less well understood than its function in cell–matrix adhesions. Developmental studies indicate vinculin is important for cell–cell adhesion. Indeed, mice lacking vinculin die at embryonic day 10.5 [29]. These mice have severe developmental abnormalities, including neural tube closure defects and a failure of the hearts to properly fuse [29]. Both phenotypes can be explained by improper cell–cell adhesion. Similarly, sudden death occurs in the first three months of life in 49% of mice with a cardiomyocyte-specific deletion of vinculin [86]. Prior to the onset of death, ultrastructural analysis of the hearts from these mice revealed abnormal adherens junctions [86]. These observations illustrated vinculin localization to cell–cell junctions is critical for proper physiology.

How vinculin is recruited to cadherin junctions remains incompletely understood. For many years, it was thought α-catenin recruits vinculin. Evidence for this comes from the observation that vinculin does not localize to cell–cell junctions in cells lacking α-catenin or in hearts lacking α-catenin [87, 88]. However, cells expressing vinculin mutants unable to bind β-catenin, while retaining an ability to associate with α-catenin, do not localize to cadherin contacts. Hence, β-catenin is important [89]. In further support of this notion, vinculin does not localize to adherens junctions in cells lacking β-catenin [111]. These findings suggest that vinculin recruitment to cell–cell contacts may be a multistep process that involves both α-catenin and β-catenin. Two current hypotheses for localization include: (1) β-catenin recruiting vinculin and handing it to α-catenin, or (2) β-catenin recruiting vinculin and α-catenin stabilizing the interaction. More work is needed to resolve these possibilities.

Many of the initial studies examining recruitment of vinculin employed cells in culture that were not under force or were experiencing low levels of force. Subsequent studies showed that force plays an important role in recruitment of proteins to the cadherin adhesion complex. Vinculin is recruited to cadherin-containing sites in response myosin II-dependent contractility [90], myosin VI-dependent contractility [91] and external tension [92, 93]. Several different proteins may be involved in force-induced vinculin recruitment. These include α-catenin which undergoes a force-dependent conformational change exposing vinculin binding sites [94, 95] and Eplin, a protein whose inhibition results in a loss of vinculin from cell–cell junctions [96]. Finally, Kannan and Tang showed α-actinin recruits vinculin to cell junctions in response to tension [97]. Diminished expression of α-actinin or its upstream activator (synaptopodin) blocks tension-induced recruitment of vinculin to junctions [97]. However, it is unclear whether α-actinin directly recruits vinculin or indirectly recruits through an α-catenin–dependent interaction.

Another important determinant of vinculin recruitment to cadherin-containing adherens junctions is tyrosine phosphorylation. Our laboratory demonstrated that vinculin is tyrosine phosphorylated at Y822 in response to force on E-cadherin, but not integrins. Mutant Y822F versions of vinculin are unable to bind β-catenin and do not localize to adherens junctions, thereby suggesting that phosphorylation at Y822 may affect vinculin conformation [98]. This idea is supported by Berrtouchi et al. who used super-resolution microscopy to assay vinculin localization at cadherin-containing contacts and a FRET biosensor to assay vinculin conformation [99]. They discovered an Y822E phosphomimetic undergoes a change in FRET and repositions itself in an actin-binding layer, thereby suggesting a vinculin conformational change [99]. Hence, tyrosine phosphorylation of Y822 may affect vinculin localization by controlling its conformation state.

Functions for vinculin at cell–cell contacts are emerging. Specific deletion of vinculin from adherens junctions (while leaving its functions at cell–matrix adhesions unperturbed) decreases cell–cell adhesion and results in a loss of E-cadherin from the cell surface [89]. Vinculin binding to β-catenin is critical for this effect. In support of this notion, mutant versions of vinculin unable to bind β-catenin do not rescue E-cadherin expression at the plasma membrane [89]. This role is reminiscent of the function of vinculin in cell–matrix adhesions where vinculin anchors integrins to the cytoskeleton during focal adhesion maturation and increases the integrin residency time in focal adhesions. Hence, the role of vinculin in stabilizing adhesion receptors appears to be conserved in cell–cell and cell–matrix adhesions.

In addition to modulating adhesion, emerging evidence suggests transmitting force is another important function of vinculin in cell–cell junctions. Le Duc et al. established E-cadherin is a mechanosensor that transmits force to the actin cytoskeleton and vinculin plays a key role in this process [100]. Evidence for this stems from studies indicating mechanosensing is lost in cells lacking vinculin or expressing a mutant form of vinculin unable to be phosphorylated at Y822 [98]. However, vinculin is not likely to be the sole mediator of the E-cadherin mechanotransduction as other work suggests a role for α-catenin and/or Eplin in E-cadherin mechanotransduction and in recruiting and maintaining vinculin in cell–cell junctions in cells under tension [96].

How vinculin regulates cell–cell adhesive events remains speculative. One likely possibility is that vinculin modulates actin dynamics to establish linkages to the actin cytoskeleton. In support of this notion, Leerberg et al. found VASP binding to vinculin is necessary for vinculin to regulate junctional actin assembly in cells under force [91]. An alternative, but not necessarily mutually exclusive possibility, is vinculin stabilizes α-catenin in a conformation that binds F-actin. In this scenario, α-catenin, not vinculin, would be the major linkage between the cadherin adhesion complex and the actin cytoskeleton.

### Future directions/unanswered questions about vinculin

In this review and others, the roles of vinculin in cell–cell and cell–matrix adhesions are often considered as separate entities. However, a number of studies indicate a balance of cell–cell adhesion and cell–matrix adhesion is critical for proper development [109]. Consistent with this notion, there is considerable cross talk between the two adhesion sites with changes in adhesion/force transmission at one site affecting change at the other site (reviewed in [101, 102]). Indeed, integrin binding to the matrix strengthens cadherin-mediated adhesion [103]. Similarly, elevations in integrin-mediated traction forces are accompanied by increases in myosin-dependent tension at cadherin contacts [104, 105]. The opposite is also true–tension on cadherins affects integrins. Specifically, cells in contact with their neighbors can generate more traction forces than single cells [106, 107]. More investigation is required to understand how its activities are coordinated at the two adhesion sites.

Adhesion/force transmission at one cell–matrix adhesions does not always equate to changes in cell–cell adhesion/force transmission and vice versa. For example, increasing the density of vascular endothelial cells plated on a substratum elevates cell–cell contact and decreases cell–matrix adhesion [108]. Mechanisms for distinguishing the function of one adhesion site are not well understood. We recently discovered that the application of force on cadherins, but not on integrins, stimulates Abl tyrosine kinase activation and phosphorylation of Y822 vinculin [98]. Furthermore, the extent of Y822 vinculin phosphorylation determined the degree to which cadherins transduce force. In contrast, Y822 vinculin phosphorylation has no effect on integrin force transmission. Hence, phosphorylation is one mechanism to distinguish vinculin function at cell–cell and cell–matrix adhesions. It is highly likely that other mechanisms exist for coordinating the functions of vinculin and its binding partners. Future work is needed to identify these key regulatory pathways.
